# No Clinical Benefit of Empirical Antimicrobial Therapy for Pediatric Diarrhea in a High-Usage, High-Resistance Setting

**DOI:** 10.1093/cid/cix844

**Published:** 2017-09-26

**Authors:** Vu Thuy Duong, Ha Thanh Tuyen, Pham Van Minh, James I Campbell, Hoang Le Phuc, Tran Do Hoang Nhu, Le Thi Phuong Tu, Tran Thi Hong Chau, Le Thi Quynh Nhi, Nguyen Thanh Hung, Nguyen Minh Ngoc, Nguyen Thi Thanh Huong, Lu Lan Vi, Corinne N Thompson, Guy E Thwaites, Ruklanthi de Alwis, Stephen Baker

**Affiliations:** 1 The Hospital for Tropical Diseases, Wellcome Trust Major Overseas Programme, Oxford University Clinical Research Unit; 2 Children’s Hospital 1, Ho Chi Minh City, Vietnam; 3 Wellcome Trust Sanger Institute, Wellcome Trust Genome Campus, Hinxton, Cambridge, United Kingdom; 4 University of Medicine and Pharmacy at Ho Chi Minh City; 5 Children’s Hospital 2; 6 The Hospital for Tropical Diseases, Ho Chi Minh City, Vietnam; 7 Centre for Tropical Medicine, Nuffield Department of Clinical Medicine, Oxford University; 8 The Department of Medicine, University of Cambridge, United Kingdom

**Keywords:** nontyphoidal *Salmonella*, *Campylobacter*, *Shigella*, pediatric diarrhea, antimicrobial resistance, multidrug resistance, fluoroquinolones, disease outcome

## Abstract

**Background:**

Pediatric diarrheal disease presents a major public health burden in low- to middle-income countries. The clinical benefits of empirical antimicrobial treatment for diarrhea are unclear in settings that lack reliable diagnostics and have high antimicrobial resistance (AMR).

**Methods:**

We conducted a prospective multicenter cross-sectional study of pediatric patients hospitalized with diarrhea containing blood and/or mucus in Ho Chi Minh City, Vietnam. Clinical parameters, including disease outcome and treatment, were measured. *Shigella*, nontyphoidal *Salmonella* (NTS), and *Campylobacter* were isolated from fecal samples, and their antimicrobial susceptibility profiles were determined. Statistical analyses, comprising log-rank tests and accelerated failure time models, were performed to assess the effect of antimicrobials on disease outcome.

**Results:**

Among 3166 recruited participants (median age 10 months; interquartile range, 6.5–16.7 months), one-third (1096 of 3166) had bloody diarrhea, and 25% (793 of 3166) were culture positive for *Shigella*, NTS, or *Campylobacter*. More than 85% of patients (2697 of 3166) were treated with antimicrobials; fluoroquinolones were the most commonly administered antimicrobials. AMR was highly prevalent among the isolated bacteria, including resistance against fluoroquinolones and third-generation cephalosporins. Antimicrobial treatment and multidrug resistance status of the infecting pathogens were found to have no significant effect on outcome. Antimicrobial treatment was significantly associated with an increase in the duration of hospitalization with particular groups of diarrheal diseases.

**Conclusions:**

In a setting with high antimicrobial usage and high AMR, our results imply a lack of clinical benefit for treating diarrhea with antimicrobials; adequately powered randomized controlled trials are required to assess the role of antimicrobials for diarrhea.

## Teaser

We identified high antimicrobial usage and high antimicrobial resistance in children hospitalized with bloody and/or mucoid bacteria-associated diarrhea. There was no clinical benefit of antimicrobial therapy in children with diarrhea; adequately powered randomized controlled trials are required.

**(See the Editorial Commentary by Jones et al on pages 512–3.)**

Diarrhea remains the second most significant cause of morbidity and mortality in children aged <5 years worldwide [1]. In 2010, the global burden of diarrhea was estimated to be 1.73 billion episodes, of which 36 million were characterized as moderate or severe; 26% (9.3 million) of the severe episodes were estimated to arise in Southeast Asia [1]. Among the bacterial pathogens causing diarrhea in children *Campylobacter* spp., nontyphoidal *Salmonella* (NTS), *Shigella* spp., *Escherichia coli,* and *Yersinia enterocolitica* are the most commonly identified [2, 3]. *Campylobacter*, NTS, and *Shigella* are major contributors to the global morbidity of diarrhea and account for an estimated 7.5, 7.1, and 4.8 million disability-adjusted life years, respectively; the majority of these occur in low- to middle-income countries (LMICs) [4].

The prevalence of all-cause diarrhea in children aged <5 years in Vietnam is estimated to be 7%–11% (Multiple Indicator Cluster Survey in 2000, 2006, and 2011 [5]) and accounts for as much as 12% of all-cause deaths in this age group [6]. A large burden of diarrheal disease, combined with the lack of financial and diagnostic resources in Vietnam, means that the causative agents of most diarrheal episodes are never determined. As a consequence, antimicrobials are empirically administered to children with diarrhea based solely on clinical presentation. A previous study found that antimicrobials were prescribed in 38% of acute watery diarrhea episodes in Vietnamese children found to be associated with a viral pathogen and in 60% of cases with unknown etiology [7]. The excessive use of antimicrobials in animals and humans in Southeast Asia has led to the current antimicrobial resistance (AMR) crisis in the region, with increasing resistance against many first-line antimicrobials, including fluoroquinolones and third-generation cephalosporins, in many gram-negative pathogens across the region [8]. Therefore, a better understanding of the bacterial agents of diarrhea, their corresponding AMR profile, the impact of antimicrobial treatment on clinical outcome, and the effects of empirical antimicrobials is required.

Little has been reported regarding the disease burden and clinical management of hospitalized pediatric diarrheal diseases in Vietnam. Although NTS, *Campylobacter,* and *Shigella* have been identified as major bacterial causes of diarrhea in Vietnamese children [7, 9–11], the epidemiology, AMR profiles, treatment, and the associated outcome of these bacteria in pediatric diarrhea have not been not well described. The Vietnamese healthcare system currently follows the World Health Organization (WHO) guidelines for treatment of pediatric diarrhea. These guidelines recommend the use of low-osmolarity oral rehydration solution, zinc, and ciprofloxacin or 1 of the 3 alternatives (pivmecillinam, azithromycin, or ceftriaxone) for all patients with bloody diarrhea, irrespective of age [12, 13]. However, as the prevalence of AMR in enteric pathogens increases across the region, it is uncertain how existing guidelines correspond to circulating AMR profiles, antimicrobial treatment practices, and patient outcomes in children hospitalized with diarrhea. Therefore, we conducted a prospective multicenter cross- sectional study in Ho Chi Minh City to improve the understanding of bacteria-associated diarrhea in Vietnamese children and to assess the duration of hospital stay in diarrheal patients infected with bacterial pathogens and receiving antimicrobials.

## MATERIALS AND METHODS

### Ethics

Ethical approval for this study was provided by the ethics committees of all 3 participating local hospitals and the University of Oxford Tropical Research Ethics Committee (OxTREC No. 1045-13). Written consent from parents or legal guardians of all participants was obtained before enrollment.

### Study Design and Enrollment

This study was a prospective, observational, multicenter cross-sectional study to evaluate the etiology, epidemiology, and outcomes in children (aged <16 years) hospitalized for diarrhea. Study participants were recruited from 3 tertiary hospitals (Children’s Hospital 1, Children’s Hospital 2, and the Hospital for Tropical Diseases) in Ho Chi Minh City, Vietnam, from May 2014 to April 2016.

Children hospitalized with diarrhea, defined as ≥3 passages of loose stools within 24 hours [12] with ≥1 loose stool containing blood and/or mucus, were recruited into the study. Based on characteristics of the diarrheal stools and the duration of illness, participants were classified into 3 groups: acute nonbloody diarrhea (diarrhea with mucus, <14 days), acute bloody diarrhea (diarrhea with blood, <14 days), and persistent diarrhea (diarrhea with mucus and/or blood, ≥14 days). Children were not eligible if they had suspected or confirmed intussusception at the time of enrollment. After enrollment, a short questionnaire was completed, and a fecal sample was collected and processed within 24 hours. All enrolled patients were provided with the routine standard-of-care practices at each hospital. Treatment and proxy outcomes, including patient recovery status at 3 days after enrollment and duration of hospitalization, were recorded by clinical staff at study sites. Patient status was recorded as “recovered” if the patient had <3 passages of loose stools in the past 24 hours or “improved” if the patient had fewer episodes of diarrhea and/or less mucus and/or blood compared with status at enrollment.

### Microbiological Methods

Fecal specimens were inoculated onto MacConkey agar (MC agar; Oxoid) and xylose-lysine-deoxycholate agar (Oxoid) and into selenite broth (Oxoid) and incubated at 37°C for 18–24 hours. *Salmonella* and *Shigella* were detected based on their characteristic appearance on xylose-lysine-deoxycholate and MC agar and confirmed using matrix-assisted laser desorption/ionization time-of-flight mass spectrometry (Bruker) and API20E (bioMerieux), following the manufacturer’s guidelines. *Campylobacter* was identified using *Campylobacter* selective agar (Oxoid) under microaerophilic conditions, followed by Gram staining and microscopy.

Antimicrobial susceptibility testing was performed using the Kirby-Bauer disc diffusion method on Mueller-Hinton agar (Oxoid) for *Salmonella* and *Shigella,* and on blood agar containing 5% sheep blood for *Campylobacter* and interpreted using Clinical and Laboratory Standards Institute guidelines [14] (Supplementary Table S1). Multidrug resistance (MDR) was defined as nonsusceptibility to ≥1 agent in ≥3 antimicrobial categories listed in Supplementary Table S1. Microbiology results were reported to the collaborating hospitals within 3 days of sampling.

### Statistical Analysis

Data were analyzed using Stata (version 11; StataCorp) and R (versions 3.2.2; R Foundation for Statistical Computing) software. Figures were constructed with R software, using the ggplot2 [15] and prodlim packages. Descriptive comparisons between groups were conducted using nonparametric tests, including the Fisher exact test for categorical variables and the Mann-Whitney *U* test for continuous data. Statistical comparisons between >2 groups were conducted using the χ^2^ test and the Kruskal-Wallis test for categorical and continuous variables, respectively. Kaplan-Meier curves for length of hospital stay were compared between groups, using log-rank tests. The growth status of participating patients was assessed using the WHO global database on growth and nutrition [16], the Institute for Clinical Systems Improvement’s guidelines on preventing and managing obesity in children and adolescents [17], and the macro package for Stata software (version 11) developed by WHO.

An accelerated failure time (AFT) regression model (incorporating all study patients hospitalized for ≥1 day) was constructed in 3 steps. First, the best AFT distribution fit was identified for the dependent variable, that is, length of hospital stay. Second, 11 demographic variables (eg, sex and age), clinical symptoms of disease severity, treatment types, and MDR were tested by means of univariate analysis, using a log-normal distribution. The 11 variables were chosen because of their potential to affect duration of hospital stay. Third, a multivariate log-normal model was constructed using a stepwise backward elimination method, where variables were removed based on the likelihood ratio test (*P* < .05).

## RESULTS

### General Characteristics of Patients Hospitalized With Diarrhea

Between May 2014 and April 2016, a total of 3166 hospitalized children meeting the study criteria were recruited at the 3 study hospitals. The majority of patients were male (1945 of 3166; 61.4%), with ages ranging from 1 month to 15 years (median age, 10 months; interquartile range [IQR], 6.5–16.7 months). Patients were hospitalized for a median of 5 days (IQR, 3–7 days) with 88.7% (2808 of 3166) of patients showing improvement or resolving symptoms within 3 days of enrollment.

Patients were segregated into 3 diarrheal types: acute nonbloody (1775 of 3166; 56.1%), acute bloody (1096 of 3166; 34.6%), or persistent (295 of 3166; 9.3%) (Table 1). Owing to the clinical complexity of persistent diarrhea, statistical comparisons were conducted between the 2 acute diarrhea groups only. Patients with nonbloody diarrhea were more likely to experience vomiting than those with bloody diarrhea (66.8% vs 38.7%; *P* = .001) and dehydration (15.0% vs 2.6%, respectively; *P* = .001). A significantly greater proportion of patients with bloody diarrhea experienced abdominal pain (26.4% vs 19.3% of those with nonbloody diarrhea; *P* = .001). Systemic C-reactive protein (CRP) concentrations were significantly higher in patients with bloody than in those with nonbloody diarrhea (21.0 [IQR, 6.7–40.2] vs 7.0 [4.0–24.0] mg/L; *P* < .001).

**Table 1.** Demographic and Clinical Manifestations of Pediatric Patients Admitted With Diarrhea in Ho Chi Minh City^a^

Characteristics	Patients, No. (%)			*P* Value^c^
	Nonbloody Diarrhea (n = 1775)^b^	Bloody Diarrhea (n = 1096)	Persistent Diarrhea (n = 295)	
Sociodemographic				
Male sex	1135 (63.9)	633 (57.8)	177 (60.0)	.001
Age, median [IQR], mo	11.5 [7.6–18.5]	9.0 [5.9–15.5]	5.5 [4.0–8.2]	<.001
Growth^d^				
Obese or overweight	184 (11.0)	83 (8.3)	24 (8.7)	.02
Wasted or severely wasted	176 (10.5)	107 (10.6)	25 (9.1)	.69
Clinical symptoms				
Episodes per day, median [IQR], No.	7 [5–10]	7 [5–10]	7 [5–10]	**…**
Moderate or severe dehydration^e^	267 (15.0)	28 (2.6)	9 (3.1)	.001
Abdominal pain	343 (19.3)	289 (26.4)	50 (17.0)	.001
Fever (≥37.5°C) at enrollment	1137 (64.1)	615 (56.1)	83 (28.2)	.001
Vomiting	1185 (66.8)	424 (38.7)	101 (34.4)	.001
Hematology				
Neutrophil count, median [IQR], 10^3^/μL	4.4 [2.6–7.0]	4.1 [2.4–6.7]	2.5 [1.6–3.9]	.01
CRP, median [IQR], mg/L	7.0 [4.0–24.0]	21.0 [6.7–40.2]	5.0 [3.3–7.0]	<.001
Stool culture positive				.7
*Campylobacter*	100 (5.6)	155 (14.1)	2 (0.7)	<.001
*Salmonella*	179 (10.1)	289 (26.4)	10 (3.4)	<.001
*Shigella*	39 (2.2)	41 (3.7)	1 (0.3)	.02
Treatment				
Low-osmolarity oral rehydration solution	1740 (98.0)	956 (87.2)	260 (88.1)	.8
Intravenous rehydration	278 (15.7)	48 (4.4)	15 (5.1)	.001
Antimicrobials	1392 (78.4)	1079 (98.4)	226 (76.6)	.001
Fluoroquinolones^f^	739 (53.1)	920 (85.3)	140 (61.9)	.001
Zinc	1586 (89.4)	962 (87.8)	276 (93.6)	.20
Probiotics	1395 (78.6)	627 (57.2)	200 (67.8)	.001
Outcome				
Hospital stay, median [IQR], d	5 [3–6]	4 [3–6]	9 [4–10]	.003
Improved or recovered after 3 d^g^	1624 (91.5)	961 (87.7)	223 (75.6)	.008

Abbreviations: CRP, C-reactive protein; IQR, interquartile range.

^a^Data represent No. (%) unless otherwise specified.

^b^All children with nonbloody diarrhea had mucus in stools.

^c^ *P* values represent comparisons between nonbloody and bloody diarrhea using Fisher exact test for categorical data or Mann-Whitney *U* test for continuous data.

^d^Obese: weight for length *z* score >3 standard deviations [SDs] in children aged <24 months; body mass index (BMI) for age *z* score >3 SDs in children aged ≥24 months. Overweight: weight for length *z* score >2 SDs in children aged <24 months; BMI for age *z* score >2 SDs in children aged ≥24 months. Wasted: weight for length *z* score ≤2 SDs in children aged <24 months; BMI for age *z* score ≤2 SDs in children aged ≥24 months. Severely wasted: weight for length *z* score ≤3 SDs in children aged <24 months; BMI for age *z* score ≤3 SDs in children aged ≥24 months [18].

^e^Dehydration classified as described by Basaleem and Amin [19].

^f^Percentage of those receiving antimicrobials. Fluoroquinolones included ciprofloxacin and norfloxacin.

^g^Condition was described as “recovered” if patient had <3 passages of loose stool in the past 24 hours or “improved” if patient had fewer episodes of diarrhea and less mucus and/or blood than at enrollment.

We cultured the 3 key bacterial diarrheal pathogens in Vietnam (ie, NTS, *Campylobacter,* and *Shigella*), isolating 816 pathogens from 804 patients (11 coinfections) and stratified clinical manifestations and treatment data by these organisms. At least 1 of these bacteria was isolated from the fecal specimens of 44.3% with bloody diarrhea (485 of 1096) and 17.9% (318 of 1775) with nonbloody diarrhea (*P* < .001) (Table 1). Overall, NTS was the most frequently isolated of the 3 bacterial pathogens from the diarrheal children, accounting for 15.1% (478 of 3166) of all diarrheal cases, followed by *Campylobacter* and *Shigella*. *Shigella* infections were more common in older children (median age, 3 years) and were associated with more severe symptoms. However, children with *Shigella* infections recovered more rapidly than those infected with NTS or *Campylobacter* (Supplementary Table S2).

### Antimicrobial Usage for Treatment of Hospitalized Diarrheal Diseases

We also recorded the treatment regimens of the enrolled patients, which included oral rehydration solution, intravenous rehydration, zinc, probiotics, and antimicrobials. More than 90% of patients were administered oral rehydration solution, and >80% were given zinc supplementation. The use of antimicrobials within this population was high, with 85.2% of patients (2697 of 3166) receiving empirical antimicrobial treatment after admission to the hospital and before a bacterial culture result was obtained. Fluoroquinolones were the most commonly used class of antimicrobials (1799 of 2697; 66.7%). Differences in standard-of-care treatment were observed between patients with bloody versus nonbloody diarrhea (Table 1); antimicrobials were more regularly administered to patients with bloody diarrhea than to those with nonbloody diarrhea (*P* < .001). Antimicrobials, specifically fluoroquinolones, were commonly (>70%) prescribed before an etiological diagnosis in those eventually found to be infected with C*ampylobacter*, *Salmonella,* or *Shigella* (Supplementary Table S2).

### Antimicrobial Susceptibility

**Figure 1.**
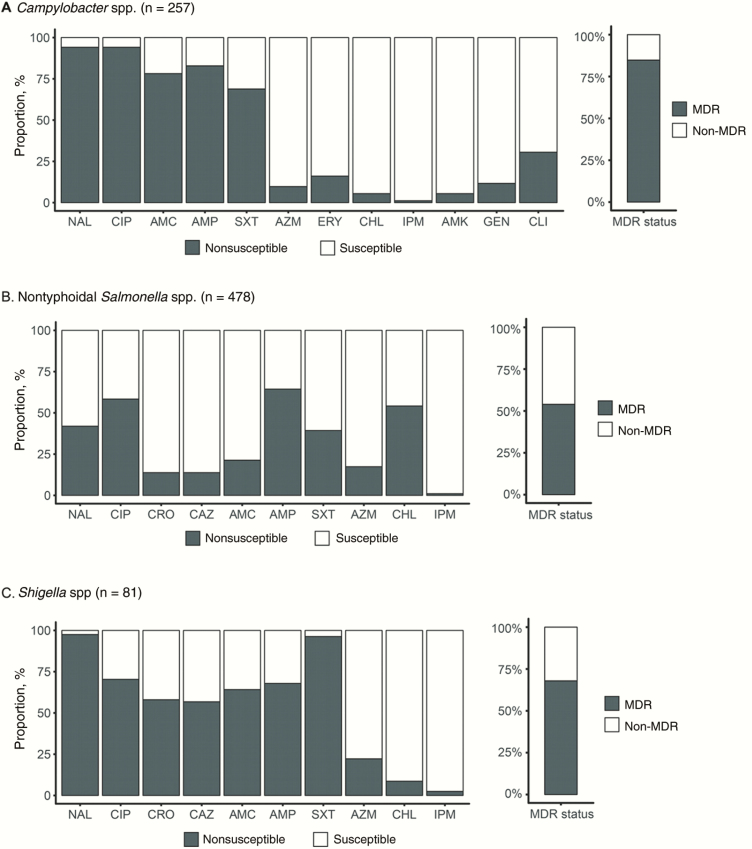
The antimicrobial resistance profiles for isolated *Campylobacter* spp. (*A*), nontyphoidal *Salmonella* spp. (*B*), and *Shigella* spp. (*C*), showing antimicrobial susceptibility and multidrug resistance (MDR), defined as nonsusceptibility to ≥1 agent in ≥3 antimicrobial categories). Bar graphs shows proportion of organisms exhibiting nonsusceptibility (dark gray) to nalidixic acid (NAL), ciprofloxacin (CIP), ceftriaxone (CRO), ceftazidime (CAZ), amoxicillin-clavulanic acid (AMC), ampicillin (AMP), trimethoprim-sulfamethoxazole (SXT), azithromycin (AZM), chloramphenicol (CHL), amikacin (AMK), gentamicin (GEN), erythromycin (ERY), clindamycin (CLI), and IPM ( ).

C*ampylobacter*, *Salmonella,* and *Shigella* isolates displayed a high prevalence of nonsusceptibility against many of the screened antimicrobials (Figure 1). Notably, a high proportion of *Campylobacter,* NTS, and *Shigella* exhibited nonsusceptibility against ciprofloxacin: 94.2% (242 of 257), 58.4% (279 of 478), and 70.4% (57 of 81), respectively. In addition, 56.8% of *Shigella* (46 of 81) and 13.8% of NTS (66 of 478) isolated were nonsusceptible to the third-generation cephalosporins, ceftriaxone and ceftazidime. A smaller fraction of the *Campylobacter,* NTS, and *Shigella* isolates also exhibited resistance against azithromycin: 9.7% (25 of 257), 17.4% (83 of 478), and 22.2% (18 of 81), respectively. The majority of the organisms (65.4%; 531 of 816 isolates) were categorized as MDR. The prevalence of MDR was highest within the *Campylobacter* isolates (218 of 257; 84.8%), with 3 isolates also exhibiting nonsusceptibly to imipenem. The MDR prevalence in NTS and *Shigella* was 53.9% (258 of 478) and 67.9% (55 of 81), respectively (Figure 1).

### Diarrheal Disease Outcome

**Figure 2.**
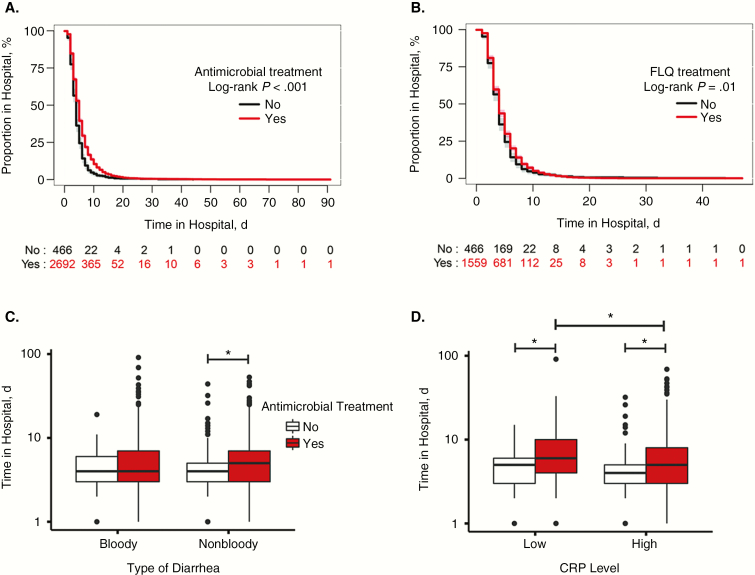
Effect of antimicrobial treatment on clinical outcome. *A*, *B,* Kaplan-Meier curves show days in the hospital for diarrheal children treated with antimicrobials (*A*) or specifically, fluoroquinolones (FLQs) (*B*). *C*, *D,* Effect of antimicrobial usage on the length of hospital stay by different diarrheal types (*C*) and blood C-reactive protein (CRP) concentration (5 mg/L cutoff) (*D*). Statistical comparisons for categorical variables were conducted using the Kruskal-Wallis test, where *.05 < *P* < .01, and *** *P* < .001. Log-rank tests were used to compare Kaplan-Meier curves for length of hospital stay between groups.

We assessed the effect of antimicrobial treatment on 2 proxy disease outcome measures, clinical outcome (ie, improved/recovered) at 3 days after enrollment and the duration of hospital stay. More than 80% of patients showed improvement or had recovered at 3 days after enrollment, regardless of antimicrobial treatment (Supplementary Figure S1). However, those given an antimicrobial, specifically a fluoroquinolone, had a longer hospital stay than those not receiving an antimicrobial (*P* < .001 and *P* = .01, respectively) (Figure 2). Notably, the duration of hospital stay did not differ significantly between those receiving and those not receiving an antimicrobial among patients with bloody diarrhea (Figure 2C). However, antimicrobial treatment in those with nonbloody diarrhea was significantly associated with a longer hospital stay (median [IQR] hospital stay for antimicrobial vs no antimicrobial use, 5 [3–7] vs 4 [3–5] days; *P* < .001; (Figure 2C). Similarly, antimicrobial treatment in patients with low CRP levels (≤5 mg/L) was significantly associated with an increased hospital stay, compared with patients with high CRP levels (>5 mg/L) (*P* < .001; Figure 2D).

**Figure 3.**
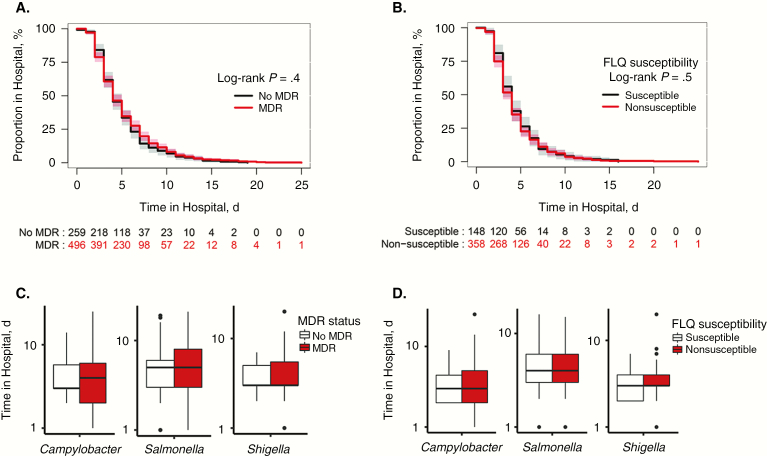
Effect of antimicrobial resistance on clinical outcome. *A, B,* Kaplan-Meier curves for length of hospital stay in diarrheal children treated with either antimicrobials or fluoroquinolones (FLQs) and stratified by multidrug resistance (MDR) (*A*) or FLQs resistance (*B*) profile of the isolated bacteria. *C, D,* Effect of MDR (*C*) and FLQ resistance (*D*) on the length of hospital stay in diarrheal children infected with *Campylobacter*, nontyphoidal *Salmonella,* and *Shigella*, while being treated with antimicrobials or FLQs, respectively. Statistical comparisons for categorical variables were conducted using the Kruskal-Wallis test. Log-rank tests were used to compare Kaplan-Meier curves for length of hospital stay between groups.

We then stratified all patients by antimicrobial treatment, and within those treated with antimicrobials we compared disease outcome (ie, duration of hospitalization) between those infected with MDR or non-MDR organisms and between those infected with fluoroquinolone-susceptible or nonsusceptible organisms. In patients empirically treated with any antimicrobial or a fluoroquinolone, >90% (526 of 564) and 70% (361 of 515) were infected with an MDR or a fluoroquinolone nonsusceptible organism, respectively. However, regardless of the MDR status and fluoroquinolone nonsusceptibility of the infecting organisms, no differences in the duration of hospitalization were observed among patients treated with antimicrobials (Figure 3). Comparable findings were observed for recovery status at 3 days after enrollment (Supplementary Figure S1).

An AFT multiple regression model was considered to be an appropriate method for describing how each of the adjusted variables multiplicatively alters the duration of hospitalization. Therefore, an AFT was constructed to investigate the effect of antimicrobials on the duration of hospital stay, adjusting for age, disease severity, and other prescribed treatments. After adjustment for age, diarrhea presentation, and dehydration, antimicrobials were associated with a significant increase in the duration of hospital stay of diarrheal patients by a time ratio of 1.32 [1.24–1.41]. Finally, infection with an MDR organism was found to not significantly prolong hospitalization (*P* = .55; Table 2).

**Table 2.** Univariate and Multivariate Analysis of Diarrheal Symptoms and Treatment on Diarrheal Disease Outcome (Length of Hospital Stay) Using an Accelerated Failure Time (Log-Normal) Model

Variable	Univariate Model			Final Multivariate Model^a^		
	β Value	TR (95% CI)^b^	*P* Value	β Value	TR (95% CI)^b^	*P* Value
Age group						
0–6 mo	1	1	…	1	1	…
7–12 mo	−.075	0.93 (.88–.98)	.01	−.106	0.90 (.85–.95)	<.001
13–60 mo	−.188	0.83 (.78–.88)	<.001	−.231	0.79 (.75–.84)	<.001
>60 mo	−.381	0.68 (.59–.79)	<.001	−.445	0.64 (.56–.74)	<.001
Sex						
Female	1	1	…	1	1	…
Male	.017	1.02 (.97–1.07)	.44	…	…	…
Diarrhea type						
Nonbloody	1	1	…	1	1	…
Bloody	−.059	0.94 (.90–.98)	.01	−.122	0.89 (.84–.93)	<.001
Fever	−.047	0.95 (.88–1.03)	.26	…	…	…
Dehydration	.193	1.21 (1.13–1.31)	<.001	.179	1.20 (1.11–1.29)	<.001
Abdominal pain	−.049	0.95 (.90–1.01)	.10	…	…	…
Probiotic treatment	.067	1.07 (1.02–1.12)	.005	…	…	…
Zinc treatment	.043	1.04 (.97–1.12)	.23	…	…	…
Rehydration	.081	1.08 (.99–1.18)	.07	…	…	…
Antimicrobial use	.229	1.26 (1.18–1.34)	<.001	.279	1.32 (1.24–1.41)	<.001
MDR	−.027	0.97 (.89–1.06)	.55	…	…	…

Abbreviations: CI, confidence interval; MDR, multidrug resistance; TR, time ratio.

^a^Adjusted β and TR values from the multivariable log-normal analysis.

^b^TRs >1 indicate an extended hospital stay; TRs <1, a decreased stay.

## DISCUSSION

Moderate-to-severe diarrhea has a significant healthcare burden in Vietnamese children [20]. Although previous observational studies of diarrhea in Vietnam have described some of the epidemiological features, the bacterial causes, and their associated AMR profiles, these studies have focused chiefly on children with acute watery diarrhea [7]. Little is known about the epidemiology and clinical management of bloody and/or mucoid diarrhea in Vietnam. Therefore, we aimed to address this paucity of data by enrolling >3000 of children hospitalized with diarrheal. This large sample size not only enabled the isolation of >800 enteric pathogens, but it also provided data regarding antimicrobial usage in medical practice and outcome. Furthermore, this study investigated the clinical role of AMR in a relevant population empirically prescribed antimicrobials at presentation to the hospital.

AMR in pathogenic bacteria, including those associated with diarrhea, is a global public health problem [21, 22]. Data from Vietnam highlight the increasing trend in diarrheagenic bacteria of AMR to the current first-line antimicrobials, such as fluoroquinolones and third-generation cephalosporins. Moreover, despite recent increases in fluoroquinolone resistance in Asia and beyond, current guidelines still endorse the use of this class of antimicrobials to treat bloody diarrhea [23–25]. Many of the organisms isolated during this investigation were also nonsusceptible to other (nonfluoroquinolone) antimicrobials, including some “last resort” choices, such as imipenem. 

In comparison to estimates from other industrializing countries, we observed a similar or elevated prevalence of NTS exhibiting nonsusceptibility to third-generation cephalosporins and increased nonsusceptibility to both ciprofloxacin and azithromycin [26, 27]. An extraordinarily high prevalence (~90%) of fluoroquinolone-resistant *Campylobacter* has been recently observed in other industrializing countries [28, 29]. The situation in Vietnam seems to be exacerbated by the nonsusceptibility of *Campylobacter* isolates to macrolides. The *Shigella* isolated here also had a high MDR rate; emerging MDR *Shigella* isolates with resistance to fluoroquinolones and extended-spectrum cephalosporins are now commonly reported across Asia [10, 30, 31].

The treatment of diarrhea with antimicrobials is a complex issue. Apart from the limited capability of most LMICs to confirm etiological agents associated with disease and the current complication of increasing AMR, there are conflicting data regarding the clinical efficacy of antimicrobials in reducing symptoms [33]. In line with WHO recommendations, we observed that >85% of patients hospitalized with diarrhea containing mucus and/or blood were prescribed an antimicrobial, most commonly fluoroquinolones (ciprofloxacin/norfloxacin). Our current analysis found that antimicrobial use during hospitalized diarrhea did not add benefit to supportive therapy only (ie, rehydration and zinc supplementation). 

The recovery of patients regardless of antimicrobial treatment or AMR status when treated with a first-line antimicrobial for bloody diarrhea treatment (eg, ciprofloxacin) may be explained by either the differing in vivo effects of antimicrobials or the self-limiting nature of the infections. In addition, in diarrhea with less pronounced inflammation (indicated by the absence of blood and/or a low CRP level), the use of antimicrobials was associated with a prolonged hospital stay. These observations support previous findings in studies of NTS and *Campylobacter,* where antimicrobial treatment did not provide a clinical advantage and sometimes even caused harm (compared with placebo) in decreasing the duration of symptoms [34, 35]. The routine antimicrobial treatment may also affect the transmission of diarrhea-causing bacteria by increasing fecal carriage and consequently spreading AMR organisms [34].

The main limitation in the present study is that this investigation was observational, with patients receiving standard-of-care treatment, which made it difficult to assess whether our observations were biased by more severe cases being prescribed antimicrobials. Furthermore, the duration of hospital stay and recovery of patients at 3 days after enrollment were proxy measures of clinical outcome, and we cannot discount that some children may have been discharged before the cessation of symptoms.

In conclusion, bacteria associated with pediatric diarrhea in southern Vietnam displayed an extensive AMR profile, thereby emphasizing the significance of etiological diagnosis of diarrhea in LMICs. Our key finding was that antimicrobial treatment was not associated with a reduction in diarrheal symptoms and even prolonged hospital stay in some groups. Therefore, we urge that adequately powered randomized controlled trials be conducted to better assess the potential benefits of antimicrobial therapy for treatment of diarrhea. These data will become essential for controlling antimicrobial usage during the present AMR crisis.

## Supplementary Data

Supplementary materials are available at *Clinical Infectious Diseases* online. Consisting of data provided by the authors to benefit the reader, the posted materials are not copyedited and are the sole responsibility of the authors, so questions or comments should be addressed to the corresponding author.

## Supplementary Material

Supplementary Materials

## Notes

***Acknowledgments.*** We acknowledge all members of the enteric infections group at Oxford University Clinical Research Unit (OUCRU) and the study teams at Hospital for Tropical Diseases (Infectious Pediatric Ward B), Children’s Hospital 1 (Gastrointestinal Ward), Children’s Hospital 2 (Gastrointestinal Ward), and the Clinical Trial Unit and Data Management Centre (OUCRU). Importantly, we also thank the enrolled children and their parents whose consent made this study possible.

***Financial support.*** This work was supported by the Wellcome Trust and the Royal Society (grant 100087/Z/12/Z). S. B. is a Sir Henry Dale Fellow, jointly funded by the Wellcome Trust and the Royal Society.

***Potential conflicts of interest.*** All authors: No reported conflicts of interest. All authors have submitted the ICMJE Form for Disclosure of Potential Conflicts of Interest. Conflicts that the editors consider relevant to the content of the manuscript have been disclosed.
