# Supplementary material for: No Clinical Benefit of Empirical Antimicrobial Therapy for Pediatric Diarrhea in a High-Usage, High-Resistance Setting
Source: Clin Infect Dis. 2017 Sep 26;66(4):504–11. doi: 10.1093/cid/cix844 (PMC5850041; doi:10.1093/cid/cix844)

**Supplementary Materials for**

**No clinical benefit of empirical antimicrobial therapy for pediatric diarrhea in a high usage, high resistance setting**

Vu Thuy Duong, Ha Thanh Tuyen, Pham Van Minh, James I Campbell, Hoang Le Phuc,
Tran Do Hoang Nhu, Le Thi Phuong Tu, Tran Thi Hong Chau, Le Thi Quynh Nhi ,
Nguyen Thanh Hung, Nguyen Minh Ngoc , Nguyen Thi Thanh Huong, Lu Lan Vi,

Corinne N Thompson, Guy E Thwaites, Ruklanthi de Alwis and Stephen Baker ^1,7,8*^

* Corresponding author: Professor Stephen Baker, The Hospital for Tropical Diseases, 764 Vo Van Kiet, Quan 5, Ho Chi Minh City, Vietnam. Tel: +84 89241761; Fax: +84 89238904; Email: sbaker@oucru.org

**Supplementary Materials include:**

Supplementary Table 1

Supplementary Table 2

Supplementary Figure 1

**Supplementary Table 1**. Zone diameter interpretive standard according to CLSI guidelines and multidrug resistance determination

| Test/ Report group | Antimicrobial Agent |  | Disk Content | Zone Diameter Interpretive Criteria (nearest whole mm) ^a^ | | | Note |
| --- | --- | --- | --- | --- | --- | --- | --- |
|  |  |  |  | **R** | **I** | **S** |  |
| **Penicillins** | | | | | | | |
| A | ampicillin | AMP | 10μg | ≤13 | 14-16 | ≥17 |  |
| **β-lactam/ β-lactamase inhibitor combinations** | | | | | | | |
| B | amoxicillin-clavulanate | AMC | 20/10μg | ≤13 | 14-17 | ≥18 |  |
| **Cephems (cephalosporins III)** | | | | | | | |
| B | ceftriaxone | CRO | 30μg | ≤19 | 20-22 | ≥23 |  |
| C | ceftazidime | CAZ | 30μg | ≤17 | 18-20 | ≥21 |  |
| **Carbapenems** | | | | | | | |
| B | imipenem | IMP | 10μg | ≤19 | 20-22 | ≥23 |  |
| **Aminoglycosides ^b^** | | | | | | | |
| A | gentamicin | GEN | 10μg | ≤12 | 13-14 | ≥15 |  |
| B | amikacin | AMK | 30μg | ≤14 | 15-16 | ≥17 |  |
| **Macrolides ^c^** | | | | | | | |
| A | azithromycin | AZI | 15μg | ≤12 | - | ≥13 | except *Shigella flexneri* |
| A | azithromycin | AZI | 15μg | ≤15 | - | ≥16 | for *Shigella flexneri* |
| A | erythromycin ^d^ | ERY | 15μg | ≤13 | 14-22 | ≥23 |  |
| **Quinolones and fluoroquinolones** | | | | | | | |
| B | ciprofloxacin | CIP | 5μg | ≤15 | 16-20 | ≥21 | except *Salmonella spp.* |
| B | ciprofloxacin | CIP | 5μg | ≤20 | 21-30 | ≥31 | for *Salmonella spp.* |
| O | nalidixic acid ^c^ | NAL | 30μg | ≤13 | 14-18 | ≥19 |  |
| **Folate pathway inhibitors** | | | | | | | |
| B | trimethoprim-sulfamethoxazole | SXT | 1.25/ 23.75μg | ≤10 | 11-15 | ≥16 |  |
| **Phenicols** | | | | | | | |
| C | chloramphenicol | CHL | 30μg | ≤12 | 13-17 | ≥18 |  |
| **Lincosamides** | | | | | | | |
| A | clindamycin ^d^ | CLI | 2μg | ≤14 | 15-20 | ≥21 |  |

There are no standard guidelines for *Campylobacter spp.*, therefore we used *Enterobacteriaceae* guidelines for tested antimicrobials apart from erythromycin and clindamycin. Multidrug resistance (MDR) was defined as non-susceptibility to ≥1 agent in ≥3 antimicrobial categories described above.

^a^ S: Sensitive; I: Intermediate; R: Resistance. Sensitive was classified as susceptible; Intermediate and Resistance were classified as non-susceptible to the tested antimicrobials

^b^ For *Salmonella spp.* and *Shigella spp.*, aminoglycosides may appear active in vitro but are known to not be effective clinically^14^. Therefore, susceptibility profiles of *Salmonella spp.* and *Shigella spp.* to aminoglycosides were not reported

^c^ Not included in MDR estimation.

^d^ Tested for *Campylobacter* isolates only and used zone diameter interpretive standard from *Staphylococcus spp.*

Supplementary Table 2. Clinical characteristics of those infected with *Campylobacter*, Non-typhoidal *Salmonella,* and *Shigella*

|  | ***Campylobacter*** | | **Non-typhoidal *Salmonella*** | | ***Shigella*** | | ***p*-value** ^b^ |
| --- | --- | --- | --- | --- | --- | --- | --- |
| Total, *N* ^a^ | 245 |  | 469 |  | 79 |  |  |
| Socio-demographic |  |  |  |  |  |  |  |
| Male, *n* (%) | 153 | (62.4) | 285 | (60.8) | 40 | (50.6) | 0.165 |
| Age in months, *median* [IQR] | 11.0 | [8.0-16.5] | 9.2 | [6.4-15.1] | 36.8 | [25.0-56.3] | **<0.001** |
| Growth ^c^ |  |  |  |  |  |  |  |
| Obese or overweight, *n* (%) | 26 | (11.2) | 49 | (10.9) | 13 | (21.0) | 0.067 |
| Wasted or severely wasted, *n* (%) | 34 | (14.7) | 59 | (13.2) | 1 | (1.6) | 0.055 |
| Type of diarrhea |  |  |  |  |  |  | 0.209 |
| Non-bloody diarrhea ^d^, *n* (%) | 95 | (38.8) | 175 | (37.3) | 38 | (48.1) |  |
| Bloody diarrhea, *n* (%) | 148 | (60.4) | 284 | (60.6) | 40 | (50.6) |  |
| Persistent diarrhea, *n* (%) | 2 | (0.8) | 10 | (2.1) | 1 | (1.3) |  |
| Clinical manifestations |  |  |  |  |  |  |  |
| Number of episodes per day, *median* [IQR] | 6 | [5-10] | 10 | [6-10] | 8 | [6-10] | **<0.001** |
| Moderate and severe dehydration, *n* (%) ^e^ | 14 | (5.7) | 26 | (5.5) | 13 | (16.5) | **0.004** |
| Abdominal pain, *n* (%) | 55 | (22.4) | 119 | (25.4) | 41 | (51.9) | **<0.001** |
| Fever (**≥**37.5°C at enrolment), *n* (%) | 151 | (61.6) | 306 | (65.2) | 57 | (72.2) | 0.666 |
| Vomit, *n* (%) | 119 | (48.6) | 196 | (41.8) | 51 | (64.6) | **<0.001** |
| Haematology |  |  |  |  |  |  |  |
| Neutrophil count (10^3^/μL), *median* [IQR] | 4.5 | [3.0-7.0] | 4.9 | [3.3-7.2] | 8.0 | [5.0-11.4] | **<0.001** |
| C-reactive protein (mg/L), *median* [IQR] | 13.4 | [6.5-31.5] | 29.0 | [10.0-48.0] | 52.0 | [24.0-82.0] | **<0.001** |
| Treatment |  |  |  |  |  |  |  |
| Low-osmolarity oral rehydration solution, *n* (%) | 208 | (84.9) | 401 | (85.5) | 59 | (74.7) |  |
| IV rehydration, *n* (%) | 16 | (6.5) | 32 | (6.8) | 15 | (19.0) | **<0.001** |
| Antimicrobials, *n* (%) | 230 | (93.9) | 440 | (93.8) | 75 | (94.9) | 0.927 |
| Fluoroquinolones, *n* (% of antibiotic treated) ^f^ | 182 | (79.1) | 326 | (74.1) | 67 | (89.3) | **0.010** |
| Zinc, *n* (%) | 226 | (89.0) | 426 | (90.8) | 68 | (86.1) | 0.256 |
| Probiotics, *n* (%) | 172 | (70.2) | 315 | (67.2) | 55 | (69.6) | 0.686 |
| Outcomes |  |  |  |  |  |  |  |
| Hospital stay in days, *median* [IQR] | 4 | [2-5] | 5 | [3-7] | 3 | [3-5] | **<0.001** |
| Improved and recovered after 3 days, *n* (%) ^g^ | 222 | (90.6) | 407 | (86.8) | 74 | (93.7) | **<0.001** |

^a^ All co-infections (which amounted to 11 co-infections) between *Campylobacter,* NTS and/or *Shigella* were removed from above analysis. Therefore, above table presents data and statistical analysis for mono-infections only.

^b^ Comparison of three bacterial pathogens using *χ*^2^ test for categorical data or Kruskal-Wallis test for continuous data

^c^ Obese: weight for length z score >3SD in children age < 24months; BMI for age z score >3SD in children age ≥24months,

Overweight: weight for length z score >2SD in children age < 24months; BMI for age z score >2SD in children age ≥24months,

Wasted: weight for length z score <-2SD in children age < 24months; BMI for age z score <-2SD in children age≥24months,

Severely wasted: weight for length z score <-3SD in children age < 24months); BMI for age z score <-3SD in children age ≥24months)^34^

^d^ All the children with non-bloody diarrhea presented mucus in stools

^e^ Dehydration classified according to Integrated Management of Childhood Illness^35^

^f^ Fluoroquinolones included ciprofloxacin and norfloxacin.

^g^ Defined as “recovered” if patient had <3 passages of loose stool in the past 24 hours or “improved” if patient had less episodes of diarrhea and less mucus and/or bloody in comparison to the condition of the patient at enrolment.

**Supplementary Figure 1.** Antimicrobial susceptibility and recovery in patients treated with antimicrobials

At 3 days post-enrollment, disease outcome was recorded as “recovered” if the patients had less than 3 passages of loose stool in the last 24 hours. The effect of antimicrobial treatment **(A)** or fluoroquinolones (FLQs) **(B)** on recovery status at 3 days post-enrollment. Also investigated the effect of antimicrobial treatment on recovery status in different diarrhea types **(C)**, blood C-reactive protein concentration (CRP) (5mg/L cuff-off) **(D)**, and isolated bacterial pathogens **(E)**. The effect of multidrug resistance (MDR) status **(F)** or FLQ susceptibility **(G)** on the recovery from diarrhea under antimicrobial or FLQ treatment, respectively. FLQ resistance: NS = non-susceptible, S = susceptible. No statistically significant differences in disease outcome were observed between groups.


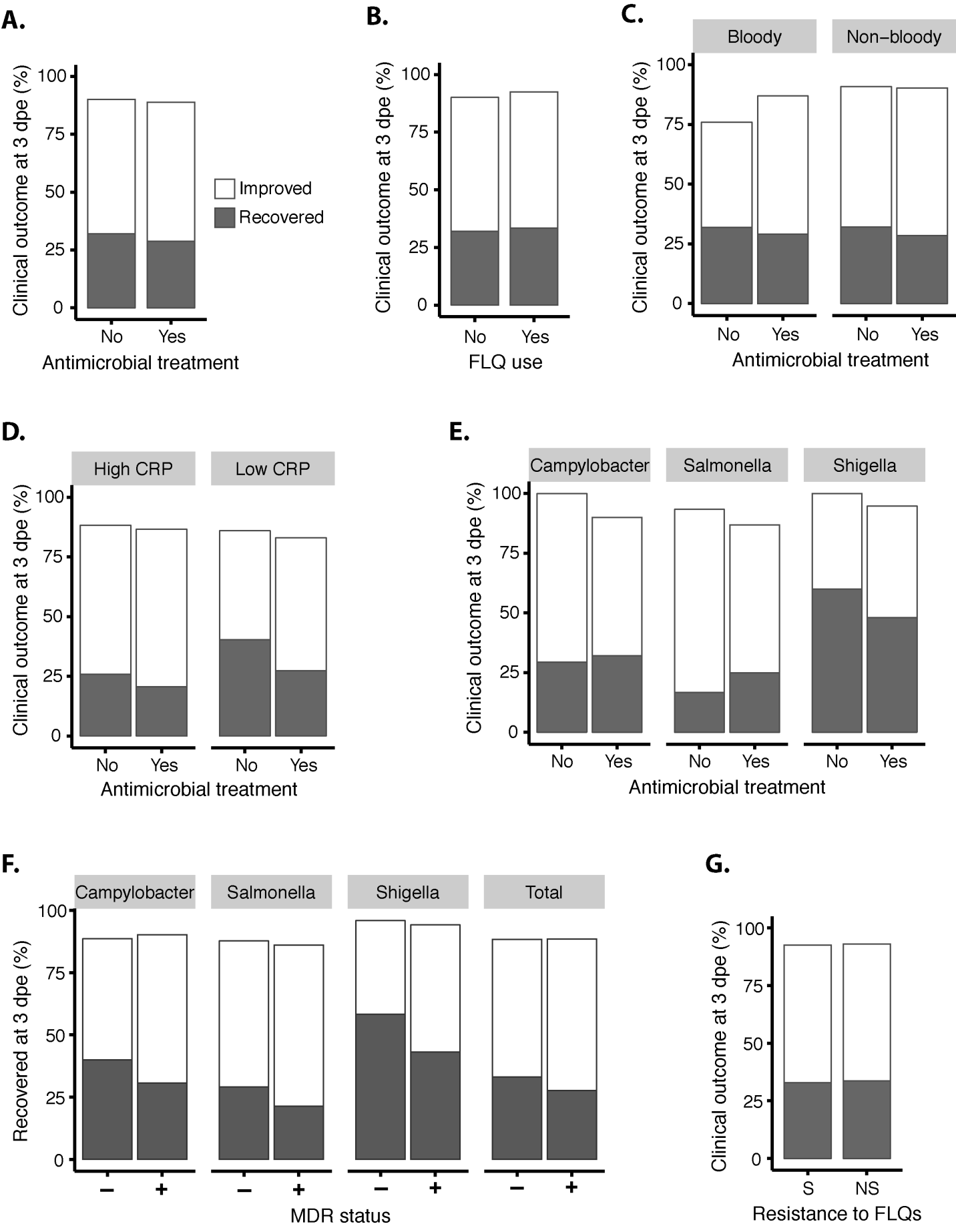

Supplement: Supplementary Materials [file cix844_suppl_supplementary_materials.docx]
